# Is it time to RE-AIM? A systematic review of economic empowerment as HIV prevention intervention for adolescent girls and young women in sub-Saharan Africa using the RE-AIM framework

**DOI:** 10.1186/s43058-020-00042-4

**Published:** 2020-06-10

**Authors:** Juliet Iwelunmor, Ucheoma Nwaozuru, Chisom Obiezu-Umeh, Florida Uzoaru, John Ehiri, Jami Curley, Oliver Ezechi, Collins Airhihenbuwa, Fred Ssewamala

**Affiliations:** 1 College for Public Health and Social Justice, Saint Louis University, Salus Center, 3545 Lafayette Avenue, Saint Louis, MO 63104 USA; 2 Mel and Enid Zuckerman College of Public Health, University of Arizona, 1295 N Martin Avenue, Tucson, AZ 85724 USA; 3 Nigerian Institute of Medical Research, 6 Edmund Crescent, Yaba, Lagos State Nigeria; 4 School of Public Health, Global Research Against Noncommunicable Diseases, Georgia State University, 140 Decatur Street SE, Atlanta, GA 30303 USA; 5 Brown School, Washington University in Saint Louis, 1 Brookings Drive, Saint Louis, MO 63130 USA

**Keywords:** RE-AIM, Economic empowerment, Adolescent girls, Young women, Sub-Saharan Africa

## Abstract

**Background:**

Economic empowerment (EE) HIV prevention programs for adolescent girls and young women (AGYW) in sub-Saharan Africa are gaining traction as effective strategies to reduce HIV risk and vulnerabilities among this population. While intervention effectiveness is critical, there are numerous factors beyond effectiveness that shape an intervention’s impact. The objective of this systematic review was to assess the reporting of implementation outcomes of EE HIV prevention programs for AGYW in SSA, as conceptualized in the RE-AIM (reach, efficacy/effectiveness, adoption, implementation, and maintenance) framework.

**Methods:**

We searched PubMed, Ovid/MEDLINE, Science Direct, Ebscohost, PsycINFO, Scopus, and Web of Science for EE HIV interventions for AGYW in SSA. Study selection and data extraction were conducted according to the PRISMA (Preferred Reporting Items for Systematic Reviews and Meta-analyses) guidelines. Two researchers coded each article using a validated RE-AIM data extraction tool and independently extracted information from each article. The reporting of RE-AIM dimensions were summarized and synthesized across included interventions.

**Results:**

A total of 25 unique interventions (reported in 45 articles) met the predefined eligibility criteria. Efficacy/effectiveness 19(74.4%) was the highest reported RE-AIM dimension, followed by adoption 17(67.2%), reach 16(64.0%), implementation 9(38.0%), and maintenance 7(26.4%). Most interventions reported on RE-AIM components such as sample size 25(100.0%), intervention location 24(96.0%), and measures and results for at least one follow-up 24(96.0%). Few reported on RE-AIM components such as characteristics of non-participants 8(32.0%), implementation costs 3(12.0%), and intervention fidelity 0(0.0%).

**Conclusions:**

Results of the review emphasize the need for future economic empowerment HIV prevention interventions for AGYW in SSA to report multiple implementation strategies and highlight considerations for translating such programs into real-world settings. Researchers should pay close attention to reporting setting-level adoption, implementation cost, and intervention maintenance. These measures are needed for policy decisions related to the full merit and worth of EE HIV interventions and their long-term sustainability for AGYW.

Contributions to the literature

- This study addresses an important gap in knowledge given the paucity of evidence regarding the extent to which existing economic empowerment as HIV prevention interventions for AGYW in SSA report on components of the RE-AIM framework.
- The RE-AIM framework is a useful evaluation framework for assessing scale-up, dissemination, or implementation of economic empowerment as HIV prevention interventions for AGYW in SSA, to ultimately enhance population impact and long-term sustainability.
- Findings from this systematic review highlight gaps in reporting of implementation outcome measures that could inform decisions around the translation and scale-up of EE HIV interventions targeting AGYW in SSA.

## Background

Across many countries in sub-Saharan Africa (SSA), adolescent girls live in a context of vulnerability and are exposed to a combination of intersecting systemic barriers based on their age, gender, education, ethnicity, socioeconomic status, and place of residence [1–3]. Every day, an estimated 1000 adolescent girls and young women aged 15–24 years are newly infected with human immunodeficiency virus (HIV) [4]. Globally, there are now 19.1 million adolescent girls and women living with HIV, of which 80% reside in sub-Saharan Africa [4]. Data from the Joint United Nations Program on HIV and AIDS (UNAIDS) estimates that three out of four new HIV infections in SSA among 15–19 years olds are among young women, and 7 out of 10 young women do not have comprehensive knowledge about HIV [5]. Additionally, the interactive effects of youth poverty and disease are particularly severe in SSA [6]. Decades of economic crisis across SSA have left millions of youth that are currently out of school unemployed [7–9]. These youth, particularly young girls, who miss out on education are more likely to engage in risk-taking behavior such as unprotected sex, transactional sex, and age-disparate sex [10–12]. Simultaneously, the population of adolescent girls and young women in SSA is expected to double from 100 million in 1990 to 200 million by 2020 [13]. This suggests a potential for new infections, and consequently a need to address the growing education and employment gap already faced by this population [14]. While there may be some challenges posed by the growing youth population in SSA, there are opportunities to optimize the demographic dividends from the “youth bulge”, where more than half of the population is younger than 20 years, to foster youth employment and economic empowerment [15]. The potential for elevated infection rates among young females in SSA demonstrates an urgent need for sustainable programs that leverage on the capabilities of young people to avert new HIV infections in adolescent girls in high HIV risk settings [16]. If not properly addressed, the mutually reinforcing crisis of poverty and disease may threaten fragile development gains. The result of which is a devastating downward spiral in human development over the next generation for millions of adolescent girls and young women in the region.

Recognizing the urgency of the crisis, considerable research has been devoted over the past two decades to developing effective strategies to prevent HIV among adolescents and young people globally [17–20]. A number of theory-based prevention approaches targeting individual-level, group, community, and structural barriers to HIV have been implemented, with some targeting girls in schools [21–28] or within their communities [29–31] and some showing evidence of efficacy or effectiveness. HIV prevention interventions also led to the development of effective approaches to combat a spectrum of other health and behavioral problems, including depression, risky sexual behaviors, pregnancy intentions, and intimate partner violence [17, 32–35]. Examples of economic empowerment interventions include microfinance, vocational skills training, business development training, micro-enterprise development, cash transfers, and savings-led asset-based programs that work to alleviate girls’ household economic hardships through the infusion of financial assets and resources [36–38]. Available evidence suggests that when implemented in conjunction with financial literacy curricula, such economic empowerment programs increase school attendance and personal savings among girls [39, 40]. Additionally, when these programs are combined with other social empowerment programs such as safe spaces, peer-support, and mentoring on female-specific issues related to health and well-being, they can increase girls’ bargaining power, decrease their financial dependence on others, and reduce engagement in sexual risk-taking behaviors [41]. Combination HIV prevention interventions that include economic empowerment activities are particularly beneficial in low-resource settings such as SSA, where adolescent girls and young women are at increased risk to engage in transactional and cross-generational sex due to limited economic assets [42–44]. Numerous studies continue to show that women who lack economic independence are less able to negotiate safe sex with partners, less able to leave an abusive relationship, and are more likely to engage in transactional sex as means of survival [45, 46]. These in turn increases their risk for HIV. Such evidence shows a strong link between economic instability and risky sexual behaviors that increase HIV risk among adolescent girls and young women in the region [43, 47].

Nevertheless, despite the increase in the number of these interventions targeting adolescent girls and young women in the region, it can take up to 17 years for these interventions to make their way to other adolescent girls underrepresented in scientific trials or in settings where its delivery could reasonably produce benefit [48, 49]. Additionally, a sizable gap remains between what is known about what works and how to effectively translate these interventions into practice [50, 51]. One potential solution is the use of implementation science, and by this, we mean the scientific inquiry into what, why, and how interventions work in “real world” settings and to test approaches to improve them [52–54]. As described by Peters and colleagues, “implementation research seeks to understand and work within real-world conditions, rather than trying to control for these conditions” [55]. It also implies working with populations that will be affected by an intervention (i.e., adolescent girls themselves serving as an advisory board), rather than selecting beneficiaries who may not represent the target population of an intervention (such as studying only in-school girls or excluding girls who have comorbidities) [54, 56, 57]. One goal of implementation science is to appropriately expand the use of interventions that have been found efficacious and as broadly as feasible in order to foster the greatest public health impact [51, 58]. The reach, effectiveness, adoption, implementation, and maintenance (RE-AIM) model is an implementation science framework for expanding interventions that have been found to be effective in research settings [58, 59]. The model focuses on the reach of the intervention to a representative proportion of the target population, the effectiveness of a program on specific outcomes, adoption of the program in a specified setting, and details of program implementation and maintenance [58, 59]. To date, there are no published studies using the RE-AIM framework to evaluate the public health impact of economic empowerment HIV prevention programs for girls, and none of the published RE-AIM studies have looked at adolescent girls and young women populations in SSA.

The present study seeks to bridge this gap between research and practice in SSA. Our objectives are twofold: (1) to review the extent to which EE HIV prevention interventions for AGYW in SSA report on implementation outcomes, as conceptualized in the RE-AIM (reach, efficacy/effectiveness, adoption, implementation, and maintenance) framework; and (2) to make recommendations for using the RE-AIM framework to advance the implementation of these interventions for girls and young women in the region. Through highlighting the reach, effectiveness, adoption, implementation, and maintenance of economic empowerment HIV interventions for adolescent girls and young women in SSA, we aim to assist researchers, practitioners, and policymakers in scaling up and evaluating new and existing economic empowerment interventions aimed at reducing the rate of new HIV infections.

## Methods

A multi-step process was used to identify, review, and analyze existing economic empowerment HIV prevention interventions targeting adolescent girls and young women in SSA using the RE-AIM framework. For the purposes of this review, economic empowerment intervention was defined as a set of economic-related actions (i.e., microfinance, cash transfers, financial literacy, savings, and asset-based programs) [60, 61] with a coherent objective to bring about change or produce identifiable HIV prevention outcomes in three broad sectors: health (i.e., girls’ overall sexual and reproductive health, HIV and sexually transmitted infections (STIs), sexual risk-taking behaviors, pregnancy, and gender-related violence), social (i.e., education-related outcomes such as school attendance, employment, mental health, future outlook, etc.), and economic (savings, asset accumulation, small business, etc.).

### Search strategy

**Fig. 1:**
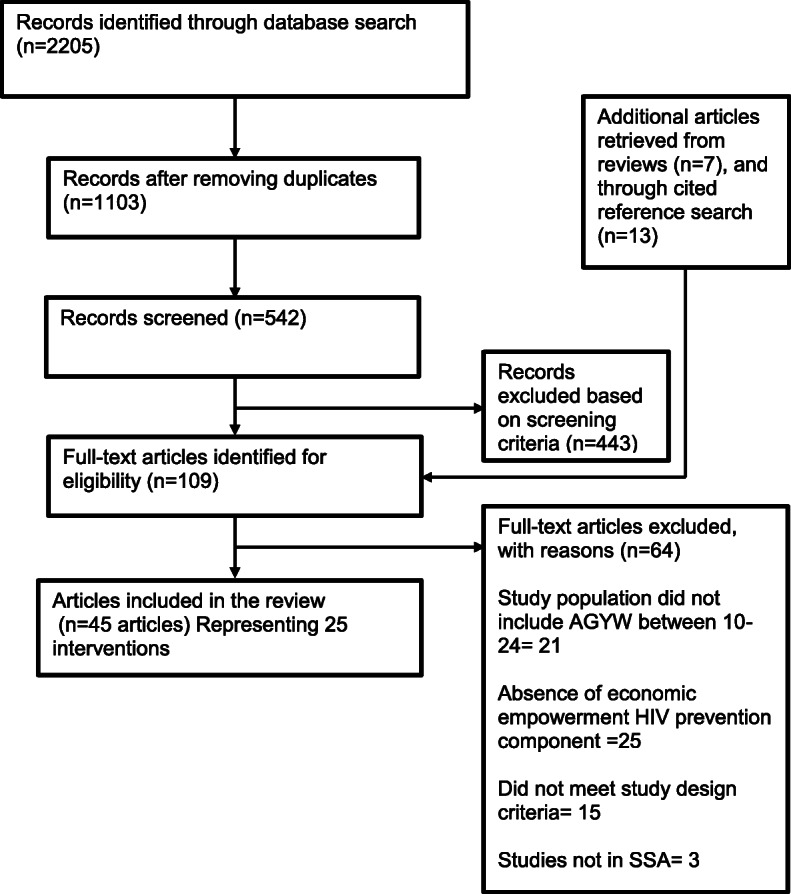
Flow diagram of the search strategy. A total of 25 unique interventions reported in 45 articles were included in the review

A systematic search of the literature was executed from October 2018 to July 2019 to locate studies published in academic journals. Figure 1 outlines the search strategy, which was reported according to Preferred Reporting Items for Systematic Reviews and Meta-Analyses (PRISMA) guidelines (see Additional file 1). Two reviewers (JI and UN) independently searched PubMed, Ovid/MEDLINE, Science Direct, Ebscohost, PsycINFO, Scopus, and Web of Science databases with the following approximate search terms: (girls or young women) AND (HIV or AIDS) AND (prevention or intervention or program) AND (economic empowerment or microfinance or cash transfers or savings-led programs or asset-based programs) AND (sub-Saharan Africa or country-specific terms for each SSA country). The search teams were modified for each database. A detailed search strategy for the PubMed database is provided in Additional file 2. In addition, published systematic reviews focused on economic strengthening for HIV prevention, as well as reference lists from the included studies, were searched to augment the database literature search. The titles and abstracts of potentially relevant articles were independently screened by two reviewers (JI and UN) for eligibility. The full texts of articles that met the eligibility criteria were obtained and assessed by the two reviewers (JI and UN) independently for inclusion in the review. Discrepancies in the screening process and study eligibility were discussed and addressed based on consensus between the two reviewers (JI and UN).

### Eligibility criteria

Inclusion and exclusion criteria were developed to identify original research that empirically evaluated or tested economic empowerment strategies to prevent HIV among adolescent girls and young women in SSA. Articles were eligible for inclusion if they were (a) conducted in sub-Saharan Africa, (b) described an economic empowerment intervention with outcomes related to HIV prevention, (c) targeted adolescent girls and young women aged 10–24 or interventions that were not specific to AGYW but reported separately on AGYW, (d) written in English, and (e) published between 2000 and 2019. We included studies that used intervention designs ranging from randomized control trials to quasi- and non-experimental evaluations of the interventions. Non-empirical studies (e.g., reviews, commentaries, editorials, and dissertations) and studies that did not explicitly assess the effect of economic empowerment on HIV prevention were excluded from the review.

### Data extraction

For studies meeting the inclusion criteria, we extracted the following data: (1) title, author, country, study objective, and design; (2) information on the intervention being evaluated, including type of economic empowerment HIV prevention intervention, and target AGYW populations; (3) components of the intervention; and (4) RE-AIM framework implementation outcomes that included (a) reach (absolute number, proportion, and representativeness of AGYW in the economic empowerment HIV prevention interventions); (b) efficacy/effectiveness (impact of the intervention on AGYW HIV prevention behaviors, including overall sexual health factors, social factors, and economic outcomes); (c) adoption (absolute number, proportion, and settings participating in the intervention, and the extent to which the settings selected are representative of settings that the target population use or visit); (d) implementation (consistency of delivery as intended, time, and cost of implementation); and (e) maintenance (extent to which a program has become part of routine practice at the organizational level or the long-term effects of a program on outcomes at the individual level) [59].

### Data analysis

Data from the articles included in this review were analyzed using narrative synthesis [62], with details on the reporting of the RE-AIM components synthesized. The articles included in this review are heterogeneous in terms of study design and measured outcomes; therefore, it was not practical to conduct a meta-analysis. To evaluate the included interventions within each dimension of the RE-AIM framework, two authors coded and scored each article independently using an adapted RE-AIM data extraction form that included a series of yes or no questions used to identify components within each of the RE-AIM dimension outcomes [63–65]. The adapted RE-AIM data extraction form is presented in Additional file 3. The form was used for calculating percentages of interventions meeting the criteria for the five RE-AIM dimensions (reach, efficacy/effectiveness, adoption, implementation, and maintenance). We summarized RE-AIM components using frequencies, proportions, and means. First, the frequencies and proportion of reported 26 components for each RE-AIM dimension were calculated separately for each study included in the review. Secondly, the average proportion of components within each RE-AIM dimension across the 25 unique interventions included in the review was calculated. The percentage and number of interventions reporting each RE-AIM dimension were reported to provide a comparable summary score across interventions.

### Risk of bias

To systematically compare the interventions, we evaluated the rigor of each intervention using the Cochrane Collaboration risk-of-bias tool [66, 67]. The tool consists of six domains: selection bias, performance bias, detection bias, attrition bias, reporting bias, and other bias [66, 67]. The risk of bias was independently rated as low, high, or unclear by two authors using the guideline for each domain. The raters discussed each domain of the assessment tool to apply consistent judgment. If ratings differed, the rationale for the rating was discussed, and the study was re-reviewed to reach consensus. The Cochrane Collaboration risk of bias assessment tool was only used to evaluate the internal validity of the interventions included in the review; no study was excluded from the review based on the risk-of-bias score.

## Results

### Study selection

The initial database search yielded 2205 potentially relevant citations based on publication titles and abstracts (Fig. 1). A total of 542 papers were retrieved for full-text review for eligibility, and 443 were excluded. The most common reasons for exclusions were the absence of an economic empowerment HIV prevention intervention targeting adolescent girls and young women aged 10–24 (*n*=46), study location outside of sub-Saharan Africa (*n*=3), or not meeting study design criteria (*n*=15). 

### Characteristics of included studies

Forty-five articles were retained in the final group of articles. Most of the articles were pulled from the electronic data searches, except for 13 articles that were identified from the manual search of reference lists. The characteristics of the final included articles are presented in Table 1. We reported on 25 unique interventions that were highlighted across the forty-five articles included in the review. All 25 interventions reported in the articles were published between 2006 and 2018, thirteen interventions were conducted in Southern Africa, nine in East Africa, and one in Central Africa, West Africa, and North Africa respectively. The majority of AGYW targeted were between the ages of 15 and 24 years. Seventeen (68%) interventions were randomized controlled trials, three (12%) were cross-sectional interventions, three (12%) were quasi-experimental designs, one (4%) observational study, and one (4%) time-series design study. Six types of economic empowerment (EE) interventions were in the included in the review: cash transfers (conditional or unconditional) [18, 41, 68, 73, 80, 86, 88, 91, 96, 97, 107], job skills or business development [70, 84, 86, 99, 108], matched savings account [71, 102], vocational skills training [75, 77, 82, 101, 105], payment of school fees and school supplies [23, 78], and loan credit [92]. Several HIV prevention outcomes were targeted, including reduction in HIV incidence [18, 22, 68, 80, 82, 97], increase in condom use [70, 73, 75, 77, 78, 83, 84, 86, 90, 92, 96, 101, 107], reduction in the number of sexual partners [70, 73, 78, 86, 101, 107], reduction in transactional sex [41, 71, 78, 82, 88, 90, 91], increase in sexual and reproductive health knowledge [71],  self-efficacy [71, 84], and delay in sexual debut [22, 73, 78, 88, 101].

**Table 1:** General characteristics of 25 interventions reported in 45 articles included in the review

Study, location	Design Description	Outcome of interest	RE-AIM dimension				
			Reach	Adoption	Implementation	Efficacy/effectiveness	Maintenance
Abdool Karim et al. (2015) [68]; Humphries et al. (2017) [69], South Africa	Cluster RCT to evaluate the impact of a cash-incentivized prevention intervention to reduce HIV infection Intervention: Cash-incentivized prevention intervention to reduce HIV infection	To increase HIV testing, reduce incidence of HIV	Study participants: 3217 adolescents; 53% females (1705) Age range, 13–24 for females (median: 16 years). Non-participants: characteristics of non-participants were not reported	Program delivered by trained field staff	Cash incentives of up to US$175 over 2 years, conditional on their participation in a life skills program, passing grades in 6 months of academic exams, and acceptance of an HIV test	After 2 years, CCTs reduced HSV-2 incidence by 30% (*p* = 0.007). Among boys, those in the incentive group had a 40% lower incidence of HSV-2 than controls (*p* = 0.042), while girls had a 24% lower incidence of HSV-2 compared to controls (*p* = 0.035). The reduction in HSV-2 infection was greater the higher the CCT amount. The number of HIV infections (75) was too small to detect a difference between intervention and control.	Individual level: Follow-up at 12 and 24 months to assess individual behavior change participants) Program level: Indicators of program level maintenance were not reported
Adoho et al. (2014) [70], Liberia	RCT comparing economic, empowerment, and health outcomes between participants and controls Intervention: Empowerment of Adolescent Girls and Young Women (EPAG) part of a larger Adolescent Girls Initiative (AGI) administered by the World Bank with support from the Nike Foundation and the Governments of Australia, the UK, Norway, Denmark, and Sweden. The intervention provided 6 months of livelihoods and life skills training (in either a Job Skills or Business Development Services track) and 6 months of follow-up support to facilitate self or wage employment for young girls (16–27 years) in Liberia	Promote safe sexual behavior	Study participants: 2042 females; Age range, 16–27 (mean, 23 years) not enrolled in school. Non-participants: Reasons for not participating in the program included (1) they were back in school, (2) they had moved to a distant location, (3) they were seriously ill, (4) they had found full-time work, (5) they were not interested or able to make such a big-time commitment, or (6) they could not be located despite numerous efforts.	Program delivered by four NGOs selected by the Liberian Ministry of Gender and Development	Empowerment of Adolescent Girls and Young Women (EPAG) provided 6 months of livelihoods and life skills training (in either a Job Skills or Business Development Services track) and 6 months of follow-up support to facilitate self or wage employment.	Among participants, there was no significant reduction in the number of sexual partners or increase in condom use as a result of the intervention. There was also no difference in these outcomes between the treatment and intervention arms. Attrition rate: 20% at 6-months follow-up Cost: $1200 for the Business Skills Track; $1650 for the Job Skills Track	Individual level: follow-up at 6 months to assess individual behavior change (sexual behavior) Program level: indicators of program level maintenance were not reported
Austrian and Muthengi (2014) [71]; Muthengi (2014) [72], Uganda	Cluster RCT to assess the impact of Adolescent Girls Empowerment Program on demographic, reproductive and health outcomes Intervention: Adolescent Girls Empowerment Program that includes safe space, health vouchers and savings account	Improve sexual and reproductive health outcomes (HIV knowledge, HIV testing, and knowledge of contraceptives) and increase in economic assets	Study participants: 4661 adolescent girls; age range, 10–19 Non-participants: characteristics of non-participants were not reported	Program implemented in partnership with key stakeholders: safe spaces with YWCA Zambia; Health Vouchers with Ministry of Community Development, Mother and Child and Savings Program with National Savings and Credit Bank and Making Cents International. Program uptake participants: 30% attended 52+ meetings	There were 3 arms: arm 1: safe spaces only; arm 2: safe spaces and health voucher; and arm 3: safe spaces, health vouchers, and savings account.	No difference between program and control with social safety nets, gender norms at 24 months. AGEP also had no impact on HIV prevalence or incidence. AGEP however improved sexual and reproductive health knowledge, improved self-efficacy, improved saving behaviors, and decreased transactional sex (for girls who were sexually active at the start). Attrition rate, 18%	Individual level: follow-up at 12 months and 24 months to assess individual behavior change among participants Program level: indicators of program level maintenance were not reported
Baird et al. (2012) [73]; Baird et al. (2013) [74], Malawi	Cluster RCT comparing conditional cash transfers (CCT) recipients versus unconditional cash transfer (UCT) recipients and non-recipients Intervention: Conditional cash transfers (CCT)	Decrease prevalence/incidence of HIV and herpes simplex virus 2 (HSV-2)	Study participants: 1706 never married young women; age range, 13–22 (schoolgirls and school dropouts) Non-participants: individuals who did not want to get tested for HIV	A local NGO implemented the CCT program	CCT (based on at least 80% school attendance) and UCT participants received some money from 1–5/month and their parents received some money from 4–10/month for 2 years	Overall, 2 years after the program ended, among school girls, neither CCTs nor UCTs had any long-term effect on HIV prevalence, onset of sexual activity, risky sexual behaviors such as having older partners or use of condoms, and the following sexual behaviors: sexual debut, age at first sex, number of sexual partners, condom use, and age of sexual partners. Among school dropouts, CCTs initially delayed the onset of sexual activity, but 2 years after the end of the program, 97% of this cohort is sexually active. CCTs did not lead to long-term changes in condom use or age at first sex among baseline dropouts. Attrition: 15.7% among school dropouts and 12.5% among school girls.	Individual level: follow-up at 6 months, 12 months, 24 months to assess individual level change Program level: indicators of program level maintenance were not reported
Bandiera et al. (2012) [75]; Bandiera et al. (2018) [76], Uganda	Cluster RCT to evaluate the effects of the Empowerment and Livelihoods for Adolescents (ELA) program Intervention: empowerment and Livelihoods for Adolescents (ELA) program	To increase HIV- and pregnancy-related knowledge and condom use	Study participants: 4800 adolescent girls. Age range, 14–20 years. Mean age, 16 years Non-participants: not explicitly stated. However, authors explain that distance to program location may have impacted participation.	Program implemented by NGO, BRAC Uganda by trained mentors or professional staff. Program uptake by participants, 21%	ELA combined the provision of life skills to reduce risk behaviors and vocational skills training to start small income-generating activities	After 2 years, among those sexually active, routine condom use increased by 25% (*p* < 0.05) and the number of girls reporting having sex unwillingly dropped from 21% at baseline to under 4% (*p* < 0.01). The intervention group also had a 26% lower rate of fertility over 2 years (*p* < 0.05). Attrition rate: 18% at 24 months’ follow-up Program costs: year 1: US$365,690; year 2: US$232,240 Individual participant cost: $17.9	Individual level: follow-up at 12 and 24 months to assess individual level behavior change Program level: authors alluded to the continuation of the program beyond study timeline. The program was expanded to include a microfinance component that provides financial support for microenterprise
Bazika (2007) [77], Congo	Cross-sectional survey and FGDs to understand how involvement in IGAs is associated with HIV risk Intervention: income-generating activities related to trade and craft apprenticeships	Increase condom use	Study participants: 372 young people; age range, 15–24 Non-participants: characteristics of non-participants were not reported	Implemented by local authorities Program uptake by participants: not stated	IGAs consisting mostly of “trade and craft apprenticeships”	Approximately 25% of the youth were involved in IGAs; 5% of all participants reported sexual intercourse with a new partner without a condom, which was significantly lower among those currently involved in IGAs (*p* < 0.01); however, higher levels of unprotected sex were reported by youth involved in agriculture.	Individuals level: follow-up at 4 years after intervention implementation to assess individual level change. Program level: the program was discontinued 3 months after implementation
Cho et al. (2018) [78], Kenya	Cluster RCT to test whether keeping orphan adolescents in school reduces HIV risk Intervention: involved providing school fees, school uniforms, and nurse’s visits to monitor absenteeism	Reduction in HIV and herpes simplex virus 2 (HSV-2) incidence	Study participants: 835 orphaned adolescents; age range, 11–20; mean age, 15 years; 48% were adolescent girls (401) Non-participants: one individual was not interested in the study. However, more details on characteristic of non-participants were not provided	Program implemented by trained research staff.	The intervention arm received payment of school fees, school uniforms, and nurse’s visits to monitor absenteeism	After 3 years, school support reduced the med-likelihood of engaging in transactional sex. High (AOR = 0.49, *p* = 0.03) and increased VMMC among males (AOR = 1.66, *p* = 0.04), but no differences were seen in sexual debut, age at first sex, number of sexual partners, or condom use between intervention and control participants. The study was underpowered to detect a difference on HIV or HSV-2 incidence between arms. Attrition rate: 10%	Individual level: follow-up at 12, 24, and 36 months to assess individual level change Program level: indicators of program level maintenance were not mentioned
Cluver et al. (2016) [79], South Africa	Prospective observational study with random sampling to assess the relationship between receipt of social services (“cash,” “cash plus care,” or “no support”) and HIV risk behaviors Intervention: Intervention provided “cash” defined as household receipt of a child support grant or foster care grant, school feeding, and/or food gardens; “cash plus care” adds receipt of teacher social support and/or positive parenting	Reduction in HIV incidence	Study participants: 2668 adolescent boys and girls; age range, 12–18; mean age, 14 years; 56% were adolescent girls (1494) Non-participants: characteristics of non-participants were not reported	Program implemented by trained research staff	“Cash” defined as household receipt of a child support grant or foster care grant, school feeding, and/or food gardens; “cash plus care” adds receipt of teacher social support and/or positive parenting.	Child-focused grants, free schooling, school feeding, teacher support, and parental monitoring were independently associated with reduced HIV-risk behavior incidence (OR 0.10–0.69). For example, girls predicted past-year incidence of economically driven sex dropped from 11% with no interventions to 2% amongst those with a child grant, free school, and good parental monitoring. Similarly, girls’ incidence of unprotected/casual sex or multiple partners dropped from 15% with no interventions to 10% with either parental monitoring or school feeding and to 7% with both interventions. Attrition rate: 3%	Individual level: follow-up at 12 months to assess individual level change Program level: indicators of program level maintenance were not reported
de Walque et al. (2012) [80]; de Walque et al. (2014) [81], Tanzania	RESPECT RCT to assess the effectiveness of Conditional Cash Transfers (CCTs) on prevention of STIs Intervention: RESPECT-provided conditional cash transfers	Reduction in risky sexual behaviors, reduce incidence of HIV, herpes simplex virus 2, and syphilis	Study participants: 2399 males and females; age range, 18–30; mean age range, 27 years;females, 50% (1199) Non-participants: the authors noted that some participants explicitly refused to participant in the study and some declined. However, detailed reasons and characteristic of these non-participants were not stated.	Program implemented by trained research staff.	CCTs of US$10 (low-value) or US$20 (high-value) per testing round conditioned on testing negative for 4 curable STIs every 4 months (3 testing rounds in 12 months)	At 12 months, the high-value CCT arm had a lower risk of combined prevalence of any of the four STIs compared to controls (aRR = 0.73, *p* < 0.05) and compared to the low-value arm (aRR = 0.69, *p* < 0.05). At end line, the combination of syphilis prevalence and new cases of HIV and HSV2 were not different between study arms. One year after the end of the intervention, both the high and low value CCTs lowered the risk of testing positive for any one of the 7 STIs (0.799 and 0.818, respectively, *p* < 0.05). Only the low-value arm significantly lowered the prevalence when looking only at the 4 STIs on which the CCT was conditioned (RR = 0.766, *p* < 0.05), and only the high-value arm significantly reduced the prevalence when looking at HIV/HSV/syphilis. Results were sustained 12 months post-intervention for males, but not females. There were no significant differences in self-reported sexual risk behavior at 24 months. Attrition rate: 6.4% attrition at 12 months and 9.3% attrition at 24 months	Individual level: follow-up at 12 and 24 months to assess individual level change Program level: conditional cash transfer and other components of the intervention were discontinued after 1 year of implementation
Dunbar et al. (2010) [82]; Dunbar et al. (2014) [83], Zimbabwe	Individual RCT to compare the effects of the Shaping the Health of Adolescents in Zimbabwe (SHAZ!) intervention on structural factors and sexual risk behaviors Intervention: Shaping the Health of Adolescents in Zimbabwe (SHAZ!) intervention on structural factors and sexual risk behaviors	Increase correct HIV knowledge, increase condom use	Study participants: 315 HIV- female, out of school orphans; age range, 16–19; mean age, 18 years Non-participants: individuals who did not return for enrollment. However, the authors did provide additional details on the characteristic of these individuals. Participants who returned to school, relocated or where influenced by partners to not participate in the study.	Program implemented by trained research staff	SHAZ! Intervention consisted of (1) reproductive health services; (2) life skills, gender, and HIV education; (3) financial literacy education and a choice of 6-month vocational training course; and (4) integrated social support and adult mentoring. The control arm received components 1 and 2 only.	After 2 years, within the intervention arm, there Med- were statistically significant reductions in high transactional sex (IOR = 0.64, *p* < 0.05), and increases in condom use with current partners (IOR = 1.79, *p* < 0.05) compared to baseline, but these were not significantly different from the results in the control group. Sexual debut also did not differ between arms. Unintended pregnancy was marginally significantly lower in intervention arm (AHR = 0.61, *p* = 0.06). Intervention participants also had a greater reduction in the experience of violence over time (AHR = 0.10, *p* = 0.06). The study was not powered to detect differences in HIV and HSV-2 incidence. Attrition: 19% at 24 months	Individual level: follow-up at 6, 12, 18 and 24 months to assess individual level change Program level: indicators of program level maintenance were not reported
Erulkar and Chong (2005) [84]; Hall et al. (2006) [85], Kenya	Longitudinal (pre-post intervention) study of Tap and Reposition Youth (TRY) participants and matched controls to assess changes in vulnerabilities and risk behaviors Intervention: Tap and Reposition Youth (TRY) - combined training on business management and reproductive health, group savings, and formal microcredit to individual group members’ contingent on other members’ timely repayment	Increase condom use and sexual and reproductive health/HIV knowledge, increase ability to negotiate issues related to sexual behavior, increase economic assets (earning, savings, household assets)	Study participants: 444 out-of-school adolescent females; age range, 16–22 Non-participants: characteristics of non-participants were not reported	Program delivered by project officers and mentors	The TRY intervention combined training on business management and reproductive health, group savings, and formal microcredit to individual group members contingent on other members’ timely repayment	At program exit (after < 1 year to 3 years), 80.3% High of TRY participants were able to refuse sex with their partner, compared to 71.6% of controls (*p* < 0.05), though TRY girls were significantly more likely to insist on condom use compared to controls (61.7% vs. 49.3%, *p* < 0.01). There was no significant difference in the likelihood of having used a condom at last sex between the two groups, though both arms experienced a decrease from baseline levels. Attrition rate: 32% at end line (36 months follow-up)	Individual level: follow-up at 12, 24 and 36 months to assess individual level change Program level: indicators of program level maintenance were not reported
Goodman et al. (2014) [86], Kenya	Stratified-random, cross-sectional survey to assess differences among three program cohorts (those involved for 4 months, over 1 year, and over 2 years) in a range of outcomes, including sexual practices. Some families received cash transfers. Some families received cash transfers Intervention: The intervention provided vocational training, group income-generating activities (IGAs), and provision of business start-up kits	Increase condom use, increase financial literacy, increase economic assets (earnings, savings)	Study participants: 707 OVC-headed households (aged 13 to 25); mean age 19 years; 66% females (467) Non-participants: characteristics of non-participants were not reported	Program implemented by community stakeholders and trained social workers	Three-year intervention grouping 20–40 families together for vocational training, group income-generating activities (IGAs), and provision of business start-up kits. They also received weekly group trainings on business, health, hygiene, and agriculture. Some families received cash transfers.	Among females, those in higher cohorts had fewer sex partners (*p* = 0.03) and greater condom use at last sexual encounter (*p* = 0.015). Among males there was no significant difference in number of sexual partners or condom use. Attrition rate: figure not reported	Individual level: follow-up at 12, 24, and 36 months to assess individual level change Program level: authors alluded to the continuation of the program and implementation in four other sub-Saharan African countries
Hallfors et al. (2011) [22]; Hallfors et al. (2015) [23]; Luseno et al. (2015) [87], Zimbabwe	Cluster RCT to assess the effects of the school subsidies (school fees, uniforms, schools’ supplies, and school helpers) on HIV risk behaviors Intervention: The intervention provided payment of school fees, uniforms, supplies, and a school-based female teacher to serve as a helper to assist with attendance monitoring and assist with attendance problems	Reduce school drop-outs, reduce unintended pregnancy, decrease age of sexual debut, and promote gender equity	Study participants: 328 orphan girls (aged 14–21 years); mean age, 12 years. Non-participants: characteristics of non-participants were not reported	Not clearly stated, but can be inferred to be members of the research team	Payment of school fees, uniforms, supplies, and a school-based female teacher to serve as a helper to assist with attendance monitoring and assist with attendance problems	At 5 years, no differences for either HIV or HSV-2 were found by study condition. Prevalence was similar, by condition, among the never married, with a trend toward higher HIV and HSV-2 infection among the married comprehensive intervention group compared with the delayed partial intervention group. Also, fewer girls among the comprehensive intervention group reported sexual debut, marriage, or pregnancy compared with the delayed partial intervention group. Attrition rate: 3% at 12 months follow-up; 12% at 24 months follow-up	Follow up at 12, 24, 36 and 60 months. Program level: the intervention lasted for 5 years. The duration of the research trial. Program level continuation in the setting was not reported
Handa et al. (2014) [88]; Rosenberg et al. (2014) [89], Kenya	Cross-sectional data from cluster RCT participants, comparing adolescent sexual debut in households receiving the transfer and those in control households Intervention: Unconditional Cash Transfer (CT) Program for orphans and vulnerable children through the Kenyan government	Reduce age of sexual debut, increase condom use, decrease number of sexual partners and decrease engaging in transactional sex	Study participants: 1433 females (out of a total sample of 2210 Orphans and Vulnerable Children (OVC)); age range, 15–25 years Non-participants: characteristics of non-participants were not reported	Program delivered by the Children’s Department of the Ministry of Gender, Children and Social Development of the Government of Kenya	Government of Kenya’s unconditional Cash Transfer (CT) Program for OVC (KES 1500 or US$20 per month per household) paid to OVC caregivers. Eligible households received monthly CTs. There was no condition placed on receiving the CTs; however, beneficiaries were told that they were expected to use the money for the care and development of the OVC resident in the household Frequency: cash was paid bimonthly to participants’ caregivers Duration: 4 years (2007–2011)	The rate of sexual debut was 38% in the CT group vs. 44% in the control (*p* = 0.001); the reduction in odds of sexual debut for CT recipients was 31%. The effect size was larger for females (AOR = 0.58) than males (AOR = 0.74), but not significantly so. Other sexual risk behaviors (engaging in transactional sex) were not statistically significantly different between the study arms. Attrition rate: 17% (between baseline assessment and first follow-up in 2009 (24 months follow-up)), 5% (between 2008–2011 (48 months follow-up)) Cost: the program budget for FY 2011/22 is KES 3.5 billion, of which 31% were from general tax revenues, 37% from development loans, and 31% from foreign aid donations.	Individual level: first follow-up at 2 years and 4 years to assess individual level change Program level: indicators of program level maintenance were not reported
Jewkes et al. (2014) [90], South Africa	Shortened interrupted time-series study to assess the effects of the intervention on HIV risk, IPV, economic, and social outcomes Intervention: Stepping Stones—the intervention provided training on livelihood strengthening through finding work or establishing a business, combined with HIV, gender, and violence prevention training	Increase household assets and promote safe sex negotiation	Study participants: 122 out-of-school young women (from a total of 232 out-of-school young people mostly under 30 years) Age range, 17–34 years Non-participants: characteristics of non-participants were not reported	Program delivered by trained facilitators from an NGO called Project Empower	Training on livelihood strengthening through finding work or establishing a business, combined with HIV, gender, and violence prevention training Intervention group: frequency—10 sessions of Stepping Stones and 11 sessions of Creating Futures Duration: 3 h bi-weekly for 12 weeks	After 58 weeks, for women there was a significant reduction in the experience of sexual IPV from 9.8% at baseline to 3.6% (*p* = 0.033), though for men there was no change in perpetration of sexual IPV. For women, there were positive but not statistically significant changes in condom use at last sex and engagement in transactional sex, while there was no change in these metrics for men Attrition rate: acknowledged but figure was not reported	Individual level: 28 weeks post-baseline and second follow-up 58 weeks post-baseline to assess individual level change Program level: indicators of program level maintenance were not reported
Khoza et al. (2018) [91], South Africa	Qualitative data collection with a sub-sample of participants in a pilot RCT (*N* = 120 adolescents) of 3 CT strategies to explore the consequences of CTs on adolescents Intervention: *CHANGE* Study—monthly cash transfers provided to participants to promote uptake of sexual risk reduction services in clinics	To promote visits to clinics for sexual reproductive health education, services related to family planning and contraception, HIV counseling and testing, HIV risk assessment, and HIV risk reduction counseling	Study participants: 49 adolescents Age range, 16–18 years Non-participants: characteristics of non-participants were not reported	This was not specified	The 3 CT strategies: (1) unconditional monthly payments of 280 ZAR (US$20) for 6 months; (2) monthly payments of 280 ZAR for 6 months, conditional on 80% school attendance; and (3) and a single payment of 280 ZAR conditional on a once-off clinic visit involving sexual reproductive health education, services related to family planning and contraception, HIV counseling and testing, HIV risk assessment, and HIV risk reduction counseling.	In interviews 6 months after the receipt of CTs and up to 12 months after the end of the intervention, some girls mentioned that CTs were protective against transactional sexual relationships. Attrition rate: not reported. May not be applicable for the qualitative study	Individual level: follow-up 6 and 12 months to assess individual level change Program level: indicators of program level maintenance were not reported
Kim et al. (2009) [92]; Pronyk et al. (2006) [93]; Kim et al. (2007) [94] ; Pronyk et al. (2008) [95], South Africa	Cross-sectional study of randomly selected matched clusters to compare associations between IPV, sexual risk behaviors, economic well-being, and empowerment between three clusters: villages exposed to IMAGE (group-based microfinance with 12-month gender and HIV training curriculum), villages exposed to microfinance (MF) only, and control villages Intervention: IMAGE—intervention with microfinance fir AIDS and gender equity. Combined intervention of group-based microfinance with gender and HIV training curriculum, Sisters for Life	Condom use, household communication about sex, communication with intimate partner about sexual matters	Study participants: 1409 female participants; 1835 people age 14–35 living with those women; and 3881 people age 14–35 living in intervention and control villages Age: 18 years and over Non-participants: authors stated that some individuals refused to participate in the study. However, the characteristic of these individuals was not reported	The microfinance component was implemented by an NGO called Small Enterprise Foundation	MF-component: groups of 5 women served as guarantors for each loan, and all 5 must repay their loans before they qualify for more credits. The loan centers met fortnightly to repay loans, apply for additional loan credit, and discuss business plan. Frequency: loan center meetings every 2 weeks Duration: individual borrowing and repayment of loans over 10 or 20-week cycle IMAGE incorporated a participatory gender-focused learning program called Sisters-for-Life into the MF-component. Frequency of the Sisters-for-Life component: phase 1 consisted of ten 1-h trainings and the phase 2 was where participants engaged youths and men in the community through community mobilization. Duration: 12–15 months	At 24 months, participants in MF-only group showed an improvement in all nine indicators of economic well-being, including household asset value, ability to repay debts, and ability to meet basic household needs compared to the control group. Likewise, compared to the control group, participants in the IMAGE group showed improved all indicators of economic well-being, as well as in empowerment (e.g., greater self-confidence, autonomy in decision-making), IPV (including reduction in past-year experience of IPV) and HIV risk behavior (including increased condom use). There was no difference between MF-only and IMAGE in improving economic well-being among participants. However, IMAGE showed great effects on improving empowerment, IPV and HIV risk behaviors among participants. After 2 years, participants in the intervention group experienced significantly less IPV in the previous 12 months compared to controls (RR = 0.45, *p* < 0.05). The intervention had no effect on the rate of unprotected sexual intercourse at last occurrence with a non-spousal partner for young people in the households of participants, or for young people living in participant villages. There was also no difference in HIV incidence among young people in intervention and comparison villages Attrition rate: 20% at 2 years follow-up; 40% at 3 years follow-up	Individual level: follow-up at 24 months and 36 months to assess individual level change Program level: the intervention was completed at the 3-year study period. However, sustainment of intervention beyond study period was not reported.
Kohler and Thornton (2012) [96], Malawi	RCT to assess the effects of two levels of CCTs on sexual risk behaviors CCTs of K500 or K2000 (USD 4 or 16) for individuals or K2000 or K4000 (USD 16 or 32) for couples were given conditional on maintaining HIV status (positive or negative) throughout the intervention period Intervention: The Malawi Incentives Project—the intervention builds upon the Malawi Diffusion and Ideational Change Project (MDICP) where participants were offered free door-to-door HIV testing and randomly assigned to cash incentives group. The Malawi Incentive projects provided conditional cash transfers in addition to the components of MDICP	Reduce HIV incidence, promote safe sex (condom use), HIV testing	Study participants: 1307 participants (55% females) Age range, 14–49 years Mean age, 36 years Non-participants: characteristics of non-participants were not reported	The Incentives were delivered by the organizers of the Malawi Incentives Project. This was not explicitly stated	The Malawi Incentives Project builds upon the Malawi Diffusion and Ideational Change Project (MDICP), where participants were offered free door-to-door HIV testing and randomly assigned to cash incentives groups. Frequency: participants received cash incentives at the end of the year if they maintained negative HIV status	There was no effect of the offered incentives on participants’ HIV status or self-reported reported sexual behaviors. Compared to male participants, female participants who received CCTs were 6.7% points less likely to engage in risky sex. Among the male participants, receipt of CCTs showed an increased likelihood of risky sex. Attrition rates: 17% among the entire sample; 16% among HIV negative participants	Individual level: follow-up at 2 years to assess individual level change Program level: indicators of program level maintenance were not reported
Nyqvist et al. (2015) [97]; Nyqvist et al.(2018) [98], Lesotho	Parallel 3—group RCT to examine the impact of a financial incentive lottery program on HIV incidence Intervention: A financial incentive lottery program to reduce HIV incidence	Reduction in HIV and other STI incidence	Study participants: 3029 females and males Number of females not specified Age range, 18–32 Non-participants: characteristics of non-participants were not reported	Program was delivered by an NGO	3 groups: (1) control arm, (2) intervention arms: Lottery incentive program separated into low-value lottery (individuals were eligible to win lottery prizes worth 500 malotis equivalent to US$50 every 4 months) and high-value lottery (individuals were eligible to win lottery prizes worth 1000 malotis equivalent to US$100 every 4 months). Participants’ eligibility for the lotteries organized every fourth month was conditioned on participants testing negative for syphilis and trichomoniasis vaginalis (2 curable STIs) a week prior to lottery draw Frequency: the lottery draws were conducted every 4 months for 2 years	Over 2 years, in the pooled intervention Med- group, HIV incidence was 21.4% lower high compared to the control (*p* < .05). In the high-prize arm only, HIV incidence was 28% lower compared to the control (*p* < 0.05); the low-prize arm was not significantly lower than the control. Effects of the intervention on HIV incidence were greater for women. The number of high-risk sexual acts was significantly reduced in the pooled intervention group compared to the control. Attrition rate: 5.4% at 16 months follow-up; 4.6% at 24 months follow-up	Individual level: follow-up at 16, 20, and 24 months to assess individual level change Program level: indicators of program level maintenance were not reported
O’Neill Berry et al. (2013) [99], Lesotho	Cross-sectional survey of participants in the Girls Empowerment program Intervention: Girls Empowerment program—provided entrepreneurial training to develop concrete, feasible, and bankable ideas to start their own small businesses as well as HIV/AIDS risk reduction and prevention, life skills	Increase knowledge on income-generating activities, reduce transactional sex, and promote HIV testing	Study participants: 40 girls aged 17–22 Non-participants: characteristics of non-participants were not reported	Program delivered by trained staff	Girls Empowerment Program (GEP) camp focused on providing entrepreneurial training to develop concrete, feasible, and bankable ideas to start their own small businesses as well as HIV/AIDS risk reduction and prevention, life skills.	Findings show considerable improvement in the girls’ knowledge about income-generating activities. In addition, almost half of the camp attendees participated in further entrepreneurial training and about half of these girls went on to develop small businesses.	Individual level: follow-up at 6 months and 12 months to report on participants’ businesses Program level: indicators of program level maintenance were not reported
Pettifor et al. (2016) [18]; Pettifor et al. (2016b) [100], South Africa	Individually randomized controlled trial to evaluate the efficacy of a CCT conditional on school attendance on HIV incidence compared to a control group Intervention: Conditional cash transfers to promote school attendance	Reduce HIV and herpes simplex virus 2 (HSV-2) incidence	Study participants: 2448 HIV negative females in high school Median age, 15 years Age range, 13–20 years Non-participants: characteristics of non-participants were not reported	Not specified	Young women and their parent/guardian received a monthly cash transfer of ZAR 100 (US$10) and ZAR 200 (US$20), respectively, conditional on 80% school attendance Intervention group received CCTs once a month. Duration: participants were eligible for CCTs up to a maximum of 3 years	Conditional cash transfer on school attendance did not reduce HIV incidence among study participants. There was no statistically significant difference in HIV incidence between participants who received cash transfer (1.94% per person-years) and those who did not (1.70% per person-years; hazard ratio 1.17, 95% CI 0.80–1.72, *p* = 0.42). School attendance however was found to reduce HIV acquisition, irrespective of study groups. Attrition rate: 9% at 36 months follow-up	Individual level: follow-up at 12, 24, and 36 months until study completion to assess individual level change Program level: not reported
Rotheram-Borus et al. (2012) [101], Uganda	Pilot pre- and post-intervention assessment of HIV risk behaviors among those receiving HIV education plus vocational training compared to those with HIV education only; cohort study of effects of combined intervention over time. Study participants were randomized to an immediate vocational training or delayed vocational training (4 months delay). The vocational training included hairdressing, catering, tailoring, mechanics, electronics, carpentry, cell phone repair, and welding. All participants in the two groups received an adapted Street-Smart HIV prevention program at the same time Intervention: The intervention provided HIV education (adapted Street-Smart HIV prevention program) plus vocational training. The vocational training included hairdressing, catering, tailoring, mechanics, electronics, carpentry, cell phone repair, and welding	Increase in condom use, reduction in number of sexual partners, economic assets (employment type and length)	Study participants: 100 youth Females not specified Age range, 13–23 years Non-participants: characteristics of non-participants were not reported	The project was delivered by an NGO—The Ugandan Youth Development league. The vocational training component was delivered by local artisans within the NGO	Frequency and duration: 10 session of the adapted HIV education session (Street Smart) over 10 weeks; 4–8 h, 5 days a week of vocational training.	At 4-month follow-up, there were no significant differences between the arms in average number of sexual partners, or in abstinence or 100% condom use. After 24 months, the combined intervention groups showed decreases from baseline in the average number of sex partners (2.12 to 1.12, *p* = 0.013) and increases in abstinence or 100% condom use (45% to 71%, *p* = 0.003). Attrition rate:15% at 4 months and 26% at 24 months	Individual level: follow-up at 4 and 24 months to assess individual level change Program level: indicators of program level maintenance were not reported
Ssewamala et al., (2009) [39]; Ssewamala et al. (2010) [102]; Ssewamala et al. (2010b) [26]; Ismayilova et al. (2012) [103], Uganda	Longitudinal RCT (pre-Suubi intervention (wave 1) and 10–12 months post-Suubi intervention (wave 2)) to examine the influence of Suubi Project on educational outcomes Intervention: Suubi Project—the intervention provided orphaned children with 3 component programs	Attitudes toward engaging in sexual risk-taking behavior, increase economic assets (savings), enhance educational plans and aspirations, reduce sexual risk-taking behaviors and attitudes	Study participants: 161 adolescent girls (out of 286 orphaned adolescents from 15 primary schools). Mean age, 13.5 years Non-participants: adolescents whose parents were skeptical of some aspects of the program, specifically the component of a matched savings account, which they said was too good to be true.	The workshops were delivered by students from Makerere University in Uganda. University students were trained by the research team	Suubi Project provides orphaned children with 3 component programs: (1) workshops focused on financial education, asset building, and career building; (2) mentorship from peers to reinforce learning; (3) a joint Conditional Development Accounts (CDAs) in both the child’s and caregiver’s name	Between wave 1 and wave 2, girls in the intervention group reported a 33% increase in positive educational plans while 27% of girls in the control group reported increase in positive education plans. In wave 2, girls in the intervention group reported statistically significant more positive educations plans than those in the control group (*t* (154) = 2.94, *p* < 0.01). Between wave 1 and wave 2, there was a 31% increase in the number of girls in the intervention group reporting more optimistic and higher level of confidence in achieving their educational plans, while in the control group, there was only 10% increase in these outcomes. In wave 2, girls in the intervention group reported statistically significant more confidence in achieving their educational plans than those in the control group (*t* (122) = 4.70, *p* < 0.00). Attrition rate: 9.1% at 10 months	Individual level: follow-up at 10 months to assess individual level change Program level: indicators of program level maintenance were not reported
Stark et al. (2018) [41]; Falb et al. (2016) [104], Ethiopia	RCT to assess the effectiveness of Child Development Accounts (CDAs) versus control condition on sexual risk-taking intentions Intervention: COMPASS program—the intervention provided (1) twelve 1-to-2-h workshops on assets building and financial planning for 10 months; (2) monthly mentorship program for adolescents with peer mentors on future planning; and (3) Child Development Accounts (CDAs) to reduce sexual risk-taking intentions	Increase school attendance, earnings, and reduce transactional sex exploitation	Study participants: 919 girls Age range, 13–19 years Non-participants: characteristics of non-participants were not reported	The program was implemented by the International Rescue Committee (IRC) and program sessions were delivered by young female mentors (from their late teens to 30 years)	The core component of the COMPASS program was to provide opportunities for girls to build assets to protect against and respond to violence and establish a foundation for a healthy transition to adulthood. COMPASS program frequency/duration: (1) twelve 1 to 2 h workshops on assets building and financial planning for 10 months; (2) monthly mentorship program for adolescents with peer mentors on future planning. The CDAs were matched savings account with a match rate of 2:1 as an incentive for participants to save, but with a limit (“cap”) on the maximum savings that could be matched (the match cap, in this case, was equivalent to US$10 a month). Each control condition adolescent received the usual care for orphaned children, which consisted of counseling and educational-related supplies (including textbooks).	The intervention did no impact economic and education outcomes measured in the study. Participants in the intervention group did not differ from those in the control group in school attendance or engaging in transactional sexual exploitation.	Individual level: follow-up at 10 months to assess individual level change Program level: indicators of program level maintenance were not reported
Visser et al. (2015) [105]; Visser et al. (2018) [106], South Africa	Mixed methods quasi-experimental design investigating differences in HIV risk behavior and other outcomes between former ISIBINDI participants and a control group Intervention: ISIBINDI—the program involves home visits to promote orphans and vulnerable children’s wellbeing. This includes optional components of career guidance, job empowerment, food gardens, and income-generating activities	Reduce HIV risk, reduce number of sexual partners, and to promote consistent condom use	Study participants: 604 (55% female) OVC Age range, 18–25 Non-participants: characteristics of non-participants were not reported	The program was developed by the National Association of Child Care Workers, then was implemented by community-based organizations	The core of the ISIBINDI model is home visits to promote OVC wellbeing and includes optional components of career guidance, job empowerment, food gardens, and IGAs. The ISIBINDI model develops the capacity of child and youth (CYCWs) care workers to respond directly to the needs of vulnerable children, youth, and families, particularly those affected by HIV/AIDS and poverty. CYCWs training modules comprise of 14 sessions of 6–30 h over a 2–3-year period.	12.9% of ex-participants of ISIBINDI reported HIV risk behavior compared to 19.7% of controls (*p* = 0.012). The percentage of participants that received food aid was not reported. Attrition rate: not reported	Individual level: follow-up duration not reported (there was post-intervention assessment) Program level: indicators of program level maintenance were not reported

### Quality of evidence

The quality assessment of the selected articles is reported in Table 2. The level of bias varied widely, with a range of 0.0% to 71.4% risk among the interventions. Among the interventions using quantitative methods, one of the interventions [18] was found to have a 0.0% (low) risk of bias. The risk of bias for quantitative methods ranged from 0.0% (low) [18] to 71.4% (high) [77]. Among interventions using mixed methods, one of the interventions [92] was also found to have a 0.0% (low) risk of bias. The risk of bias for mixed methods interventions also ranged from 0.0% (low) [92] to 71.4% (high) [99]. The only qualitative study in the review had a high risk of bias (71.4%) [91].

**Table 2:** Reporting on quality of included interventions (25 interventions reported in 45 papers included in the review)

	Selection bias (random sequence generation)	Selection bias (allocation concealment)	Performance bias	Detection bias	Attrition bias (incomplete outcome data)	Reporting bias (selective reporting)	Other sources of bias	% risk of bias	Comments
Abdool Karim et al. (2015) [68]; Humphries et al., (2017) [69], South Africa	Low risk	Low risk	Low risk	Low risk	Unclear	Low risk	Low risk	14.3%	Study design: quantitative (comparison of treatment and control groups)
Adoho et al. (2014) [70], Liberia	Low risk	Unclear	Low risk	Low risk	Low risk	Low risk	Unclear	28.6%	Study design: quantitative (comparison of two treatment groups to a control group)
Austrian and Muthengi (2014) [71]; Muthengi (2014) [72], Uganda	High risk	Unclear	Low risk	Low risk	Low risk	Low risk	Unclear	42.9%	Study design: mixed methods (comparison of two treatment groups to a control group)
Baird et al. (2012) [73]; Baird et al. (2013) [74], Malawi	Low risk	Low risk	Low risk	Low risk	Unclear	Low risk	Low risk	14.3%	Study design: mixed methods (pre- and post-test comparison for intervention and control groups)
Bandiera et al. (2012) [75]; Bandiera et al. (2018) [76], Uganda	Low risk	Unclear	Low risk	Low risk	Low risk	Low risk	Low risk	14.3%	Study design: quantitative (pre- and post-test comparison for intervention and control groups)
Bazika (2007) [77], Congo	Unclear	Unclear	Low risk	Low risk	Unclear	Unclear	Unclear	71.4%	Study design: quantitative (pre- and post-test assessment of intervention participants)
Cho et al. (2018) [78], Kenya	Low risk	Unclear	Low risk	Low risk	Low risk	Low risk	Low risk	14.3%	Study design: quantitative (comparison between intervention and control groups) Longitudinal study with annual repeated measures over 4 years
Cluver et al. (2016) [79], South Africa	Low risk	Unclear	Low risk	Low risk	Low risk	Low risk	Low risk	14.3%	Study design: quantitative (comparison between cash alone and integrated cash plus care intervention for HIV-risk reduction) Prospective longitudinal study
de Walque et al. (2012) [80]; de Walque et al. (2014) [81], Tanzania	Low risk	High risk	Low risk	Low risk	Low risk	Low risk	Low risk	14.3%	Study design: quantitative (pre- and post-test comparison for intervention and control groups)
Dunbar et al. (2010) [82]; Dunbar et al. (2014) [83], Zimbabwe	Low risk	High risk	Low risk	Low risk	Low risk	Low risk	Low risk	14.3%	Study design: quantitative (pre- and post-test comparison for intervention and control groups)
Erulkar and Chong (2005) [84]; Hall et al. (2006) [85], Kenya	High risk	High risk	Low risk	Low risk	Low risk	Low risk	Unclear	42.9%	Study design: quantitative (pre- and post-assessment of intervention participants) Longitudinal study
Goodman et al. (2014) [86], Kenya	Low risk	Unclear	Low risk	Low risk	Unclear	Low risk	Low risk	28.6%	Study design: quantitative (cross-sectional comparison among 3 cohorts)
Hallfors et al. (2011) [22]; Hallfors et al. (2015) [23]; Luseno et al. (2015) [87], Zimbabwe	Low risk	Unclear	Low risk	Low risk	Low risk	Low risk	Low risk	14.3%	Study design: quantitative (comparison between intervention and control groups) Longitudinal study with annual repeated measures over 3 years
Handa et al. (2014) [88]; Rosenberg et al. (2014) [89], Kenya	Low risk	Unclear	Low risk	Low risk	Low risk	Low risk	Low risk	14.3%	Study design: quantitative (comparison between intervention and control groups) Longitudinal study with repeated measures
Jewkes et al. (2014) [90],South Africa	High risk	High risk	Low risk	Low risk	Unclear	Low risk	Unclear	57.1%	Study design: mixed methods (interviews and quantitative time series design for pre- and post-intervention assessment)
Khoza et al. (2018) [91], South Africa	Low risk	Unclear	Unclear	Unclear	Unclear	Low risk	Unclear	71.4%	Study design: qualitative (using interviews)
Kim et al. (2009) [92]; Pronyk et al. (2006) [93]; Kim et al. (2007) [94]; Pronyk et al. (2008) [95], South Africa	Low risk	Low risk	Low risk	Low risk	Low risk	Low risk	Low risk	0.0%	Study design: mixed methods pre- and post-test comparison of intervention and control group)
Kohler and Thornton (2012) [96], Malawi	Low risk	Unclear	Low risk	Low risk	Low risk	Low risk	Low risk	14.3%	Study design: quantitative (pre- and post-comparison between intervention and control groups) (longitudinal study)
Nyqvist et al. (2015) [97]; Nyqvist et al.(2018) [98], Lesotho	Low risk	Unclear	Low risk	Low risk	Low risk	Low risk	Low risk	14.3%	Study design: quantitative (pre- and post-comparison of intervention and control groups)
O’Neill Berry et al., (2013) [99], Lesotho	Unclear	High risk	High risk	Low risk	High risk	Low risk	High risk	71.4%	Study design: mixed method (pre- and post-test comparison of intervention and control group, as well as follow-up observations of intervention group)
Pettifor et al. (2016) [18]; Pettifor et al. (2016b) [100], South Africa	Low risk	Low risk	Low risk	Low risk	Low risk	Low risk	Low risk	0.0%	Study design: quantitative (pre- and post-comparison of intervention and control groups)
Rotheram- Borus et al. (2012) [101], Uganda	Unclear	High risk	Low risk	Low risk	Low risk	Low risk	Unclear	42.9%	Study design: quantitative Had a delayed intervention group. Pre- and post-test comparison between immediate intervention and delayed intervention group
Ssewamala et al., (2009) [39]; Ssewamala et al. (2010) [102]; Ssewamala et al. (2010b) [26]; Ismayilova et al. (2012), Uganda	Low risk	Unclear	Low risk	Low risk	Low risk	Low risk	Low risk	14.3%	Study design: quantitative (pre- and post-comparison of intervention and control groups)
Stark et al. (2018) [41]; Falb et al. (2016) [104], Ethiopia	Low risk	Unclear	Low risk	Unclear	High risk	Low risk	Unclear	57.1%	Study design: quantitative (pre- and posttest comparison for intervention and control groups). Reported null findings that the intervention did not seem to keep the participants in school, nor influence out-of-school girls to return to school
Visser et al. (2015) [105]; Visser et al. (2018) [106], South Africa	High risk	Unclear	Low risk	High risk	High risk	Low risk	Unclear	71.4%	Study design: mixed method (quasi-experimental post-intervention assessment between intervention and control group and focus group discussions). Utilized focus group discussions to generate information on strategies to sustain ISIBINDI intervention

The most common strengths of the interventions that utilized quantitative methods were: the ability to conduct a longitudinal follow-up of study participants over time, the random selection and assignment of participants, and the reporting of descriptive intervention details. However, one of the common weaknesses was the limited use of intent-to-treat analysis, although attrition was acknowledged by the majority of the interventions [18, 22, 41, 70, 71, 75, 78–80, 83, 84, 88, 92, 96, 97, 101, 102]. For the interventions that utilized mixed-methods [71, 73, 90, 92, 99, 105], common strengths were the ability to triangulate data obtained from qualitative and quantitative methods and providing additional explanation for the quantitative data using qualitative data. The strength of the qualitative studies were the use of detailed quotes and narratives to explain study findings.

### Reporting of RE-AIM dimensions

The reporting of RE-AIM dimensions was assessed using a previously developed and validated data extraction tool that included implementation outcome components based on the RE-AIM framework [59, 63]. Across all the interventions, average reporting rates (defined here as the overall percent of components) were highest for efficacy/effectiveness ≈19(74.4%) and adoption ≈17(67.2%), followed by reach 16(64.0%), and lowest for implementation ≈9(37.3%) and maintenance ≈7(26.4%). Table 3 provides details on each of the components assessed across the RE-AIM framework and a summary of the overall percentage of interventions reporting on each of the RE-AIM dimensions. The reporting status for the 26 components for the RE-AIM dimensions per study is provided in Additional file 2.

**Table 3:** Proportion of interventions reporting RE-AIM dimensions and components

RE-AIM dimensions and components	Reporting frequency (*n* = 25)	Reporting proportion (%)
**Reach**		
Method to identify target population	24	96.0
Inclusion criteria	16	64.0
Exclusion criteria	3	12.0
Sample size	25	100.0
Participation rate	9	36.0
Characteristics of participants	25	100.0
Characteristics of non-participants	8	32.0
Representativeness	18	72.0
^a^Average of overall reach dimensions	*16.00*	*64.00*
**Efficacy/effectiveness**		
Measures/results for at least one follow-up	24	96.0
Intent to treat utilized	5	20.0
Quality-of-life measure	23	92.0
Baseline activity measured	25	100.0
Percent attrition	16	64.0
^a^*Average of overall efficacy/effectiveness dimensions*	*18.60*	*74.4*
**Adoption**		
Description of intervention location	24	96.0
Description of staff who delivered intervention	22	88.0
Method to identify target delivery agent	16	64.0
Level of expertise of delivery agent	19	76.0
Adoption rate	3	12.0
^a^*Average of overall adoption dimensions*	*16.80*	*67.2*
**Implementation**		
Intervention duration and frequency	25	100.0
Extent protocol delivered as intended	0	0.0
Measures of cost of implementation	3	12.0
^a^*Average implementation dimensions*	*9.33*	*37.3*
**Maintenance**		
**Individual-level maintenance**		
Was individual behavior assessed ≥ 6 months post-intervention	23	92.0
Was individual behavior assessed ≥ 24 months post-intervention^a^	3	12.0
Was individual behavior assessed ≥ 48 months post-intervention^a^	4	16.0
**Program-level maintenance**		
Indicators of program continuation	2	8.0
Some measure/discussion of alignment with organization/setting	1	4.0
^a^*Average of overall maintenance dimensions*	*6.60*	*26.4%*

^a^Average percent for overall 26 components within each RE-AIM dimension. The proportions are based on the 25 unique interventions included in the Review. Components were included to ensure relevance with HIV prevention health behavior change

### Reach

The average proportion reporting on the reach components was 16(64.0%). The sample size and participants’ characteristics were the most frequently reported item 25 (100%). All interventions reported on sample size, defined as the number of participants who consented to participate in the study/intervention. Of the interventions that recruited only AGYW (*n*=12) [18, 22, 41, 70, 72, 73, 75, 84, 87, 92, 99], sample size ranged from *n*=40 in the O’Neill Berry and colleagues study [99] to *n*=4800 in the study by Bandiera and colleagues [75]. In interventions that recruited other populations in addition to AGYW, the sample size ranged from *n*=46 to *n*=6576 (with the AGYW sample size being between 122 and 1705). Five (20%) interventions [77, 91, 96, 97, 101] did not explicitly report the sample size for AGYW, although it was mentioned that AGYW were included in the study. Participant characteristics included; reports on age, gender (for interventions that included other population), employment status, education attainment, and socioeconomic status (measured as household income in some interventions). The next commonly reported reach component was the method for identifying the target population for the study and this was assessed in 24(96.0%) of the studies reviewed [18, 22, 39, 41, 68, 70, 71, 73, 75, 77, 78, 80, 82, 84, 86, 88, 90–92, 96, 97, 99, 101, 105]. Most of the interventions were conducted in Southern Africa, specifically in South Africa where 7 [18, 68, 79, 90–92, 105] out of the 25(28%) interventions were located. The description of methods utilized to identify the target population varied across interventions from single-sentence descriptors to detailed reporting of the protocol used. Strategies utilized to identify the target population included using schools, youth centers, and community stakeholders. Regarding factors that foster or hinder the ability to reach the target audience, stakeholders’ engagement and school recruitment were emphasized as beneficial strategies to enhance reach.

Sixty-four percent(16) of the interventions [18, 26, 41, 70, 73, 75, 80, 83, 84, 86, 88, 90–92, 96, 97] reported study participants’ inclusion criteria. Only 3(12.0%) studies [18, 73, 80] explicitly stated participants’ exclusion criteria. Participant inclusion criteria were typically related to participants’ age, place of residence, membership (e.g., being part of the school), parental status (being an orphan), and gender. Individuals were mainly excluded if they did not meet the inclusion criteria for the interventions. Participation rate was reported in nine(36.0%) [18, 68, 70, 75, 79, 83, 90, 92, 96] of the included interventions. The participation rate ranged from 21% to 97.50%. Eighteen(72.0%) of the interventions [18, 41, 68, 70, 73, 75, 78, 80, 83, 84, 86, 88, 90–92, 97, 102, 107] reported on the representativeness of recruited study participants’ relative to the target population. This was determined based on comparing demographic characteristics (e.g., age, education level) of study participants to those of the target population. Reporting on this component allowed the researchers to assess the extent to which the intervention could be generalizable across the target population and setting. The rigor of the study design was reported as an indicator of representativeness. Interventions that utilized randomized controlled trials reported representativeness as one of the strengths of their studies. According to the RE-AIM framework, studies should describe the characteristics of participants of the target population in comparison with non-participants. Eight(32%%) of the interventions provide some form of information on the characteristics of individuals who did not participate in their study. Some of the reasons for non-participation included unavailability of individuals (e.g., going back to school and having full-time jobs), inability to complete study procedures (e.g., not wanting to test for HIV, not returning for study procedure, and not obtaining consents from parents), limited access to the study location (e.g., distance from the individuals’ residence to study site was a barrier to participating and geographic relocations) and lack of interest in the study.

### Efficacy/effectiveness

Efficacy/effectiveness was the most consistently reported RE-AIM dimension across all interventions (74.4%). Twenty four(96.0%) interventions reported on at least one post-intervention effect; 5(20.0%) interventions used intent-to-treat analyses and the remainder analyzing only data from participants who completed the intervention. All interventions included in the review included HIV prevention measures as primary outcomes. HIV prevention measures included; reduction in HIV incidence [22, 68, 79, 80], reduction in number of sexual partners [70, 73, 79, 86], condom use [70, 73, 75, 77, 78, 83, 84, 86, 90, 92, 101], decrease in transactional sex [41, 71, 78, 83, 89–91], and sexual debut [23, 73, 78, 83, 87, 88, 97]. Of the 25 interventions that measured HIV prevention outcomes, 20(80%) reported that the economic empowerment HIV prevention intervention resulted in statistically significant positive changes in HIV prevention outcomes.

Sixteen(64.0%) interventions reported their percent attrition [22, 70, 71, 75, 78–80, 83, 84, 88, 92, 96, 97, 100–102], which ranged from 5% to 40%. Attrition rates were examined in relation to participants’ loss to follow-up and non-use of the intervention [75]. Reasons for attrition included; participants’ relocation, death, change of phone number, and logistics challenges. In terms of logistics challenges, Erulkar and Chong [84] reported some delays in participants receiving their loans and accessing their savings account as a result of limited human resources which accounted for some of the attritions they faced. Some participants were concerned that they may not have access to their savings account or loans; therefore, they dropped out of the study. In addition, Bandiera and colleagues [75] examined how participants’ characteristics influence attrition between the intervention and control groups and found that married AGYW in the intervention were less likely to be tracked at follow-up. A high proportion of the interventions 23(92.0%) reported on participants’ quality of life [18, 22, 39, 41, 70, 71, 73, 75, 77, 78, 80, 82, 84, 88, 90–92, 96, 97, 99, 101, 105] and found that economic empowerment HIV prevention interventions generally improved quality of participants lives and did not have any significant negative outcomes.

### Adoption

The average proportion reporting on adoption components was 17(67.2%). Twenty-two of the interventions [22, 39, 41, 68, 70, 71, 73, 75, 78, 80, 82, 84, 86, 88, 90, 92, 96, 97, 99, 101, 105] provided some description of the staff who delivered the intervention. Interventions were delivered by a range of staff with different levels of expertise and included research assistants, community leaders, and organization staff. Staff responsibilities included delivering parts of the interventions that consisted of moderating the discussion and intervention meeting groups, distributing conditional cash incentives, training participants’ income-generating skills, and educating participants on intervention curriculum (e.g., sexual and reproductive health training, financial training, and income-generating skills and crafts). Seventy-six percent (19) of the interventions explicitly stated implementing staff level of expertise [22, 39, 41, 68, 70, 71, 73, 75, 80, 82, 84, 86, 88, 90, 92, 99, 101, 105], but for those that were not stated, it could be inferred from their job titles or their organization’s focus. Sixteen(64.0%) interventions reported on the methods used to identify staff who delivered the intervention [22, 70, 71, 73, 75, 78, 80, 82, 86, 88, 90, 92, 97, 99, 101, 105]. Intervention staff were mainly identified through their participation in the research project or collaborating organization.

The most commonly reported adoption component was the description of intervention location, reported by 24(96.0%) studies [18, 22, 39, 41, 68, 70, 71, 73, 75, 78, 80, 82, 84, 86, 88, 90–92, 96, 97, 99, 101, 105]. Intervention locations included schools, community centers, and refugee camps. These locations were identified as typical settings that the target population visit or use. Also, most of the interventions were restricted to a specific geographical area. Most of the interventions were implemented in one site. The least reported adoption component was the adoption rate. Only 3 (12.0%) interventions reported on intervention adoption rate among participants [86, 99, 101]. There were no reports on setting level adoption rates.

### Implementation

The average proportion reporting on implementation components was about 9(37.3%). All 25(100.0%) interventions reported on the format of the intervention; specifically, they provided information on intervention duration and frequency [18, 22, 39, 41, 68, 70, 71, 73, 75, 77, 78, 80, 82, 84, 86, 88, 90–92, 96, 97, 99, 101, 105]. Intervention ranged in duration from a single session to two or more (up to 14) sessions. None of the interventions explicitly reported on fidelity or the extent to which the intervention protocol was delivered as intended.

The cost of delivering the intervention was mentioned in only three (12.0%) interventions [70, 73, 75]. Implementation cost items included skills training cost [70, 75], administrative cost [73, 75], and cost of monetary incentive [73, 75]. Two interventions [70, 75] further conducted cost-benefit analyses to determine if the benefits/returns from the interventions for the participants outweighed the cost of implementing the interventions. These two interventions assessed intervention benefit based on the number of participants who participated in the income-generating component of the intervention. The authors highlighted that equipping AGYW with skills to generate sustainable income, which would in return reduce their chance of engaging in risky sexual behaviors [70, 75]. Specifically, Adoho and colleagues [70] found that the value provided by the program was equivalent to a 3 year increase in income among EE program participants. The study by Bandiera and colleagues [75] reported gains/benefits to the economic empowerment intervention in the form of delaying early marriage and childbirth and improving HIV and pregnancy-related knowledge.

### Maintenance

The average proportion reporting on maintenance components was about 7(26.4%). Among the maintenance components, individual-level indicators were reported more frequently than program-level indicators. Twenty-three (92.0%) interventions reported at least one follow-up measure, particularly the primary outcomes at 6 months [18, 22, 39, 41, 68, 70, 71, 73, 75, 77, 78, 80, 82, 84, 86, 88, 91, 92, 96, 97, 99, 101]. The longest follow-up period reported was 24 months after baseline assessment [71]. The majority of the post-intervention assessments were conducted within 12 to 24 months after completion of the intervention. There were a few interventions that had follow-up assessments beyond 24 months after intervention completion; 6 for 36 months [18, 22, 78, 84, 86] follow-up, 2 for 48 months [88, 89] follow-up, and 1 for 60 months follow-up [23].

In terms of program-level maintenance, six interventions reported [22, 75, 77, 80, 86, 92] on indicators of program level maintenance or sustainability. Only two(8%) interventions explicitly stated that the interventions were sustained beyond the study period [75, 86] For one study, the intervention was adapted to fit the context by including an additional component [75]. Two(8%) interventions were discontinued before the study period end date [77, 80], and another two(8%) ended at the completion of the study period [22, 92]. For the two interventions that were completed at the end of the study period, it was not clear if they were sustained beyond the study period.

## Discussion

The primary aim of this review was to systematically assess the implementation of economic empowerment HIV prevention programs for AGYW in SSA. This review goes beyond an assessment of intervention effectiveness to report implementation outcomes as conceptualized by the RE-AIM framework. The RE-AIM framework was used as a guideline to determine the impact of EE HIV prevention interventions for AGYW. We evaluated five key components important for the translation of research findings to practice: reach, effectiveness, adoption, implementation, and maintenance [59, 109]. These components are important in understanding the factors that influence, not only adoption, but the cost and sustainability of economic empowerment interventions as a strategy for HIV prevention among AGYW in SSA.

A total of 25 (reported in 45 papers) economic empowerment interventions among AGYW were identified, described, and evaluated based on the five RE-AIM dimensions. On average, the included interventions reported on 14(53.86%) of the 26 components that constitute the RE-AIM dimensions. Major knowledge gaps exist relating to reporting of implementation and maintenance (least reported RE-AIM dimensions) of economic empowerment HIV interventions for AGYW in SSA. Specifically, the interventions in the review mainly focused on reporting intervention-specific components (e.g., sample size, intervention location, and effectiveness), with minimal reporting of broad or system-level components such as implementation costs, program-level sustainability, and intervention fidelity. Although concerning, the underreporting of broad or system-level elements is consistent with reports from other systematic reviews using the RE-AIM framework [110–115] that also found limited reporting of these dimensions. This further confirms the previous report on the predominant focus on intervention effectiveness, with limited attention to external factors that may impact the translation of effective interventions to real-world settings. Researchers need to also focus on reporting broad or system-level measures as well as intervention-specific measures. Broad or system-level factors are critical with understanding how findings from interventions apply to local settings, population, and available resources [116]. It informs the overall relevance and appropriateness of these interventions in real-world settings, and the potential for health gains by reducing HIV incidence among AGYW in SSA. 

Reporting on intervention reach is important to inform future dissemination of interventions that have been found to be effective or efficacious towards behavior change. To scale-up economic empowerment HIV prevention intervention, there is the need to understand how to reach target populations. In this review, participants’ characteristics and sample sizes were consistently reported across interventions. This is congruent with previous reviews on HIV prevention interventions [32, 117] that reported frequent reporting of participants characteristics such as their demographics. Some of the interventions in the review specified the degree to which the  target samples were representative of the larger population. Information on the external population from which a study sample is drawn from helps to inform the generalizability of the findings to a larger population [111]. However, the characteristics of non-participants and participants, as well as the reasons for non-participation, were rarely reported in the interventions. This limits the understanding of contextual factors that may influence AGYW participation in such interventions. With scant information on characteristics of non-participants, researchers may be missing individuals who are most in need of these interventions, such as AGYW residing in remote areas, rural areas, and those with low literacy. To enhance the translation of intervention to a wider population, researchers should improve on the reporting of the characteristics of non-participants as this may extend program reach and inclusivity.

Consistent with past reviews, intervention effectiveness was the most commonly reported RE-AIM element across all interventions, with baseline activity measures reported for all included interventions [110, 111]. The outcome measures included; HIV incidence, number of sexual partners, condom use, transactional sex, and sexual debut. Findings from this systematic review highlight the impact on economic empowerment intervention on HIV reduction among AGYW, with about 19(74.7%) of the interventions reporting statistically significant improvements on HIV risk reduction measures among intervention participants compared with controls. For effectiveness analyses, only 5(20%) interventions reported using intent-to-treat analyses; this in turn may have impacted the positive effect of the intervention across the interventions. The positive effect found in these interventions were only limited to participants who were present for follow-up assessments and did not account for attrition. There were variations in the reporting of attrition rates across the interventions, and few studies provided information on reasons for attrition. Information on the reasons for attrition may help to highlight barriers or challenges that influence AGYW participation in the interventions. For instance, one of the interventions encountered some logistics challenges in the form of delays in providing loans to participants [84]. Such logistical challenges are critical information that may influence AGYW attrition and participation in HIV prevention interventions. Thus, efforts to account for factors influencing attrition are necessary for identifying barriers and challenges to AGYW continued participation in interventions.

For adoption, the description of the intervention location, staff delivering the intervention, and level of staff expertise were well documented in the reviewed interventions. However, there was minimal reporting on the methods used to enhance staff and intervention settings adoption. This is consistent with other reviews using the RE-AIM framework, where there is consistent under-reporting on methods used to enhance adoption by intervention delivery agents [111, 118]. This makes it challenging to determine what types of delivery agents may be appropriate for the optimal implementation of the intervention [111]. Furthermore, only 3(12%) interventions reported on the intervention adoption rate. Reporting of adoption rate and characteristics of participating intervention locations versus non-participating locations may help highlight components of intervention design that either hinder or foster adoption across various settings [111].

The cost of intervention implementation is an important factor in determining the translation of research findings to real-world settings. Three (12%) of the 25 interventions in the review reported on the cost of intervention delivery. The findings of the review reveal a paucity of data on the cost and cost-effectiveness of implementing economic empowerment HIV prevention interventions among AGYW. Report on delivery cost allows for effective planning to optimize the yield and reach of economic empowerment HIV prevention for AGYW [119–121]. Likewise, documenting cost-effectiveness is crucial for sustainability and large-scale dissemination of HIV prevention interventions in SSA [120]. Cost information also helps to allocate resources efficiently particularly in settings were resources are scare. This in turn may help maximize the impact of positive health outcomes among AGYW [119].

In terms of implementation, intervention duration and frequency were consistently measured across the interventions. However, none of the interventions reported on the fidelity of the study, although it is a critical measure of the internal validity of the interventions. Therefore, it is unclear if the reported intervention impact were attributed to the fidelity of the intervention or to the actual intervention components [110]. Considering the critical role of these components in enhancing the impact and scale-up of such intervention, the scarcity of evidence in this area is a concern. Future interventions should clearly specify implementation components such as fidelity to enhance the translation of these interventions to other settings and populations.

Regarding maintenance, about 7(26%) reported on this RE-AIM dimension. This is a favorable result, compared to other reviews that have reported between 0.0% and 11.0% maintenance [110, 122]. This RE-AIM dimension helps to understand the long-term maintenance of behavior change among intervention participants and the sustainability of the interventions at implementing locations. Most of the interventions measured maintenance of individual behavior at least 6 months following the completion of the intervention, with only 4 of the interventions measuring behavior at 48 months after intervention completion. While individual-level maintenance components were frequently reported, little attention was paid to the assessment of setting- and program-level maintenance components. Intervention maintenance also known as sustainability is influenced by an interplay of individual-, program-level factors and broader socio-cultural- and community-level factors, which collectively determine long-term intervention impact. Therefore, future research should address critical gaps in the assessment of intervention maintenance, and apply a more comprehensive approach in the evaluation of this implementation outcome dimension.

## Limitations

Our review has some limitations. First, our conclusions are based on the degree to which the included interventions reported on specific RE-AIM dimensions. It is possible that some of the RE-AIM dimensions were measured, but not reported in the interventions due to editorial restrictions. To address this limitation, we included all available articles on a specific intervention. Second, we did not conduct a meta-analysis. While this was not the focus of this systematic review, the heterogeneity of the included interventions and variations in HIV prevention outcomes would not have supported a meta-analysis. Third, our search strategy was limited to published articles and those available in English; this is potentially subject to selection bias. Fourth, it is worth noting that the Cochrane Collaboration risk of bias assessment tool used in assessing study quality is biased towards purely quantitative study designs and quite limited in appraising mixed and qualitative study designs. Given the limitation of this tool, it was only used to evaluate the internal validity of the interventions included in the review and not to select articles included in the review.

Nonetheless, this study has a number of strengths. First, this review was conducted with a well-constructed search strategy, created with the help of the college librarian, and was supplemented by a manual search of the reference list of included articles. Second, to the best of our knowledge, this is the first study to collate and examine the measurement of implementation outcomes among economic empowerment interventions HIV prevention for adolescent girls and young women in sub-Saharan Africa using the RE-AIM framework as a guide.

## Conclusion

Emerging evidence suggests that economic strengthening interventions can be effective in reducing adolescent girls’ and young women’s risks for HIV. RE-AIM assessment showed that economic empowerment intervention provides AGYW with skills to reduce their risk of HIV. Our findings further show that although researchers frequently reported on intervention-specific implementation science outcome components, broad or system-level implementation outcome indicators of these interventions are scarce. Considering the critical role of these implementation factors in enhancing the ultimate impact of combination economic strenghtening intervention on HIV prevention among AGYW in SSA, the scarcity of evidence is a concern. We recommend the use of RE-AIM components in future EE HIV interventions targeting AGYW, with special consideration given to factors relevant to the adoption, implementation (such as implementation cost, adoption rate, and intervention fidelity) and long-term sustainability of these interventions in SSA. We further suggest the measurement of other implementation science outcomes beyond RE-AIM indicators to provide a holistic indicator of factors and measures to promote intervention scale-up, dissemination and sustainability. Overall, the findings of this systematic review and the use of the RE-AIM framework, have the potential to accelerate the tempo of implementation and dissemination of evidence-based interventions for addressing HIV prevention among at-risk AGYW in SSA. 

## Supplementary information

**Additional file 1.** PRISMA checklist.

**Additional file 2.** Search strategy.

**Additional file 3.** RE-AIM components extraction.

## Data Availability

Articles included in this systematic review are cited in the reference list.

## Abbreviations

AGYW: Adolescent girls and young women
CCTs: Conditional cash transfer
CA: Collins Airhihenbuwa
CO: Chisom Obiezu-Umeh
FE: Fred Ssewamala
FU: Florida Uzoaru
HIV: Human immunodeficiency virus
JC: Jamie Curley
JI: Juliet Iwelunmor
JE: John Ehiri
OE: Oliver Ezechi
PRISMA: Preferred Reporting Items for Systematic Reviews and Meta-Analyses
RCTs: Randomized control trials
RE-AIM: Reach, effectiveness, adoption, implementation, and maintenance
SSA: Sub-Saharan Africa
STIs: Sexually transmitted infections
