# Supplementary material for: Is it time to RE-AIM? A systematic review of economic empowerment as HIV prevention intervention for adolescent girls and young women in sub-Saharan Africa using the RE-AIM framework
Source: Implement Sci Commun. 2020 Jun 10;1:53. doi: 10.1186/s43058-020-00042-4 (PMC7427963; doi:10.1186/s43058-020-00042-4)
Supplement: Supplementary file 2 — Additional file 2. Search strategy. [file 43058_2020_42_MOESM2_ESM.docx]

Additional File 2: Search strategy for PubMed which was modified and used in other databases

| Category | Search Terms Combined with AND |
| --- | --- |
| Age group | (adolescent[MeSH] OR young adult[MeSH] OR adolescents OR youth OR “adolescent girls” OR “young women” OR female OR girls) |
| Age | 10 to 24 |
| HIV-related outcomes | (HIV OR AIDS OR “human immunodeficiency virus” OR AIDS OR sexual OR “sexual behavior” OR “sexual activity” OR “sexual debut” AND “transactional sex”) |
| Income-generation related intervention | (“cash-transfers” OR “asset-based” OR “micro-credit” OR microcredit OR “job training” OR “income generation” OR “income-generating” OR “job skills” OR employment OR “economic empowerment” OR cooperatives OR “micro-finance” OR “microfinance” OR microfinance OR “micro-enterprise” OR “micro-enterprise” OR microenterprise OR “small business” OR “small loans” OR “micro-loans” OR microloans OR “micro-loans” OR “vocational training” OR “vocational skills training” OR “business training” OR livelihood) |
| Intervention | ("HIV interventions" OR "HIV prevention" OR "HIV risk reduction" OR programs or interventions) |
| Region/countries | ( “Africa” [MeSH] OR “Sub-Saharan Africa” OR Africa OR Cameron OR Central African Republic OR Chad OR Congo OR Democratic Republic of the Congo OR Equatorial Guinea OR Gabon OR Eastern Africa OR Burundi OR Djibouti OR Eritrea OR Ethiopia OR Kenya OR Rwanda OR Somalia OR Sudan OR Tanzania OR Uganda OR Southern Africa OR Angola OR Botswana OR Lesotho OR Malawi OR Mozambique OR Namibia OR South Africa OR Swaziland OR Zambia OR Zimbabwe OR Western Africa OR Benin OR Burkina Faso OR Cape Verde OR Cote d'Ivoire OR Ivory Coast OR Gambia OR Ghana OR Guinea OR Guinea-Bissau OR Liberia OR Mauritania OR Niger OR Nigeria OR Senegal OR Sierra Leone OR Togo) |
| Implementation outcomes | (implementation OR "implementation outcomes" OR "implementation research" OR reach OR effectiveness OR adoption OR uptake OR implementation OR maintenance OR acceptability OR appropriateness OR costs OR feasibility OR fidelity OR cost OR penetration OR sustainability)) |

Note: MeSH = medical subject heading
